# The eukaryotic translation elongation factor 1A regulation of actin stress fibers is important for infectious RSV production

**DOI:** 10.1186/s12985-018-1091-7

**Published:** 2018-11-26

**Authors:** Natale Snape, Dongsheng Li, Ting Wei, Hongping Jin, Mary Lor, Daniel J. Rawle, Kirsten M. Spann, David Harrich

**Affiliations:** 1 Faculty of Medicine, The University of Queensland Diamantina Institute, Brisbane, Australia; 2 Department of Cell and Molecular Biology, QIMR Berghofer Medical Research Institute, Qld, Herston, 4029 Australia; 3 School of Chemistry and Molecular Biosciences, University of Queensland, Qld, St. Lucia, 4072 Australia; 4 School of Biomedical Science and Institute of Health and Biomedical Innovation at the Centre for Children’s Health Research, Queensland University of Technology, Qld, Brisbane, 4101 Australia

**Keywords:** Respiratory syncytial virus, Eukaryotic translation elongation factor 1A, Didemnin B, Stress fibers, Virus replication

## Abstract

Cellular protein eukaryotic translation elongation factor 1A (eEF1A) is an actin binding protein that plays a role in the formation of filamentous actin (F-actin) bundles. F-Actin regulates multiple stages of respiratory syncytial virus (RSV) replication including assembly and budding. Our previous study demonstrated that eEF1A knock-down significantly reduced RSV replication. Here we investigated if the eEF1A function in actin bundle formation was important for RSV replication and release. To investigate this, eEF1A function was impaired in HEp-2 cells by either knock-down of eEF1A with siRNA, or treatment with an eEF1A inhibitor, didemnin B (Did B). Cell staining and confocal microscopy analysis showed that both eEF1A knock-down and treatment with Did B resulted in disruption of cellular stress fiber formation and elevated accumulation of F-actin near the plasma membrane. When treated cells were then infected with RSV, there was also reduced formation of virus-induced cellular filopodia. Did B treatment, similarly to eEF1A knock-down, reduced the release of infectious RSV, but unlike eEF1A knock-down, did not significantly affect RSV genome replication. The lower infectious virus production in Did B treated cells also reduced RSV-induced cell death. In conclusion, the cellular factor eEF1A plays an important role in the regulation of F-actin stress fiber formation required for RSV assembly and release.

**Electronic supplementary material:**

The online version of this article (10.1186/s12985-018-1091-7) contains supplementary material, which is available to authorized users.

## Introduction

Human respiratory syncytial virus (RSV) is recognised globally as the main cause of acute lower respiratory infection in young children. Respiratory syncytial virus is an enveloped, non-segmented, negative sense, single-strand RNA virus in the Pneumovirdae family [1, 2] and, like all viruses, is reliant on the host cell for replication and release. Many viruses use cellular translation factors to ensure their own proteins are produced, and to stifle the innate host defence mechanisms in order to rapidly proliferate [3]. For example, several RNA viruses including RSV have subverted cellular eEF1A for viral replication and genome synthesis [4, 5]. RNA viruses may utilize eEF1A because it is an abundant protein in the eukaryotic cell, second only to actin, which has multiple cellular functions besides its well-recognised role in protein translation. These non-canonical roles of eEF1A in cellular functions include nucleocytoplasmic trafficking, protein degradation, apoptosis, heat shock response and actin remodelling [5–7]. We previously confirmed that eEF1A is important in RSV replication, by demonstrating that downregulation of eEF1A using siRNAs in RSV-infected cells restricted the replication of viral genomic RNA and release of infectious virions [8]. We further demonstrated that eEF1A interacts directly with the RSV N protein, which suggests that eEF1A can engage and stabilise the viral genome replication complex. A similar function has been reported for the HIV-1 reverse transcription complex [9, 10].

One of the alternative functions of eEF1A is as an actin binding protein that can regulate actin stress fiber formation in a Rho/Rho kinase pathway dependent manner [11]. RSV requires actin for virus replication, and F-actin is reported to be important for viral transcription and morphogenesis. This is dependent on cellular profilin activity, which regulates F-actin formation in cells [12]. The seven subunit actin-related protein (ARP) 2/3 complex promotes the assembly of branched F-actin required for various cellular processes such as vesicle trafficking and plasma membrane protrusion during cell migration [13]. More recently, the ARP2 was identified in a genome-wide siRNA screen as important for RSV gene expression [14]. Further analysis showed that ARP2 specifically supported viral spread during filopodia formation that could shuttle virus from infected to uninfected cells [14, 15]. Filopodia are slender cellular cytoplasmic projections that contain F-actin cross-linked into bundles by actin-binding proteins [14, 16]. Given the association between eEF1A and actin, and the dependence of RSV on actin for virus replication, we investigated whether the reduced RSV replication by down-regulated eEF1A, as we have reported previously [8] was, in part, due to the effect of change in actin filament structure, in addition to the effect on RSV genome replication.

In this study we aimed to investigate if an eEF1A actin-related role is important for RSV replication. Two approaches were used to investigate the association between eEF1A, cellular actin and RSV replication: downregulation of eEF1A expression using siRNA, and treatment with an eEF1A inhibitor, Did B. Did B, a cyclic depsipeptide produced by *Trididemnum solidum*, binds to eEF1A and inhibits the eEF1A function in protein translation [4, 17]. The results demonstrated that down-regulating eEF1A using siRNA disrupted actin stress fiber formation and reduced RSV egress. Similarly, treatment with Did B at low concentrations, which do not inhibit cellular protein translation or induce cytotoxic effects, led to similar disruption of actin stress fiber formation and reduced RSV production. This study demonstrates the important roles of eEF1A in regulation of actin organization, and in RSV infectious virus production.

## Materials and methods

### Cell culture and virus stock preparation

Human epithelial carcinoma cells (HEp-2, ATCC) cells were grown in Opti-MEM media (Gibco) supplemented with 5% heat-inactivated foetal bovine serum and 1% penicillin-streptomycin (10,000 U/ml) (Gibco), incubated at 37 °C with 5% CO_2_.

A stock of RSV A2 (ATCC) was propagated in HEp-2 cells and purified through a 30/60% *w*/*v* sucrose cushion as described previously [18] and stored at − 80 °C until required. The titre of the resultant viral stock was quantified by standard immune-plaque assay. Briefly, virus suspension was diluted in a 10-fold series and used to infect HEp-2 cell monolayers, then incubated at 37 °C for two hours and overlaid with Opti-MEM /60% methyl cellulose/2% FBS/1% penicillin/streptomycin. After seven days incubation at 37 °C with 5% CO_2_, monolayers were fixed with 60% methanol/40% acetone, blocked with 5% skim milk in PBS and probed with goat-anti RSV polyclonal antibody (Virostat). RSV positive plaques were visualised with HRP-conjugated secondary antibody (Life technologies) and DAB colour developer (Sigma-Aldrich). Viral titre was calculated as plaque forming units (pfu)/ml.

### Treatment of HEp-2 cells with Did B

Didemnin B (Did B, kindly provided by the Natural Product Branch, NCI, USA) was dissolved in DMSO as a 10 mM stock and stored at − 80 °C. Confluent HEp-2 cells were treated with a range of concentrations from 0 nM to 16 nM. The same volume of DMSO was used as vehicle control. For effect of Did B on translation, HEp-2 cells were transfected with pCMV-Gluc2 plasmid and re-seeded into 24 well plates containing various concentration of Did B as described. The luciferase activity in culture supernatant was measured after 24 and 48 h of treatment using Biolux Gaussia luciferase Flex Assay kit (NEB). Subsequent experiments were conducted with a final concentration of 2.5 nM of Did B. Cell proliferation was monitored using CellTiter 96® Aqueous One Solution Cell Proliferation assay (Promega), as instructed by the manufacturer.

### RSV infection

HEp-2 cells were infected with RSV A2 at a multiplicity of infection (MOI) of 1 pfu/cell at 37 °C for four hours. Inoculum was then removed and the cells were washed with 1x PBS prior to treatment with Did B. Cells were then incubated at 37 °C and sampled at 24 and 48 h post infection (p.i.) with RSV.

### Downregulation of eEF1A using siRNA

siRNA targeting the eEF1A mRNA transcript (ID: SASI_Hs02_00331772 and SASI_Hs02_00331773, Sigma-Aldrich) or a universal negative control (siMM: SIC001) were transfected into HEp-2 cells using Lipofectamine RNAiMAX (Invitrogen, USA) according to the manufacturer’s protocol. The efficiencies of eEF1A down-regulation were examined at 24, 48, 72 and 96 h post-transfection (p.t.). For the effect of downregulation of eEF1A on RSV replication, RSV infection was performed at 48 h p.t..

### Fluorescent staining and confocal microscopy

Cells either treated with Did B, or transfected with siRNAs or infected with RSV were washed with PBS, fixed with 4% formaldehyde and permeabilised with 3% BSA/1% Triton X-100 in PBS. The cells were then blocked with 5% BSA in PBS. Actin was stained with Phalloidin (Sigma Aldrich), eEF1A was identified using anti-eEF1A (Santa Cruz) and RSV was identified using an anti-N antibody (Virostat). Fluorescent images were captured using a Leica TCS SP2 confocal scanning microscope (Leica Microsystems) with 100 × objective lenses. All images shown are maximum intensity projections. ZEN Blue Black software was used to quantify pixel colocalization of actin and eEF1A at the cortex from z-stack confocal images using identical parameters. At least 12 cells treated with 2.5 mM Did B, DMSO or untreated were analysed. The colocalization coefficient was calculated as previously described [19].

### Lactase dehydrogenase (LDH) release assay

The cellular release of lactate dehydrogenase into supernatant was measured by Promega’s CytoTox-ONE™ assay, performed as per manufacture’s protocol, with the following changes: 50 μl of cell culture supernatant, or OptiMEM™ as blank control, was added to individual wells of a 96 well plate. The same volume 50 μl of CytoTox-ONE™ reagent was added to each sample and incubated at 22 °C for 10 min, the reaction stopped with 25 μl CytoTox-ONE™ stop solution and quantified using a Clariostar ELISA plate reader at 450 nm.

### RT-qPCR for viral transcription and translation

Total RNA was extracted from cell pellets or 1 ml culture supernatant using TRIzol™ reagent (Life Technologies). The RNA was then reverse transcribed using Superscript III First-Strand Synthesis SuperMix™ kit (Life Technologies), as per manufactures protocol. Three separate reactions using different primers to generate cDNA were used for each sample; gene specific primers specific to RSV N mRNA, random hexamer or oligo-dT primers. Quantitative PCR was performed on the Qiagen RotorGene using primers specific for N cDNA or genomic cDNA of the RSV, and primers for cellular β-actin as an internal control as described previously [8].

### SDS-PAGE and Western blot analysis

Cell lysates were boiled in SDS-PAGE sample buffer and separated by 10% sodium dodecylsulfate – polyacrylamide gel electrophoresis (SDS-PAGE). Gels were electro-blotted onto a polyvinylidene fluoride (PVDF) membrane (Pall) using a semi-dry transfer system (Bio-Rad Laboratories). The membranes were blocked with 5% milk in PBS and probed with either anti-eEF1A antibody (Santa Cruz Biotechnology) or anti-β-tubulin antibody (Sigma Aldrich).

### Statistical analysis

Statistical analysis were performed using an unpaired one way ANOVA test or a Student’s T-test as indicated. Statistical significance was set at: * = *P* < 0.05, ** = *P* < 0.01, *** = *P* < 0.001 and **** = *P* < 0.0001.

## Results

### eEF1A knock-down disrupted actin stress fiber formation and reduced RSV egress

**Fig. 1:**
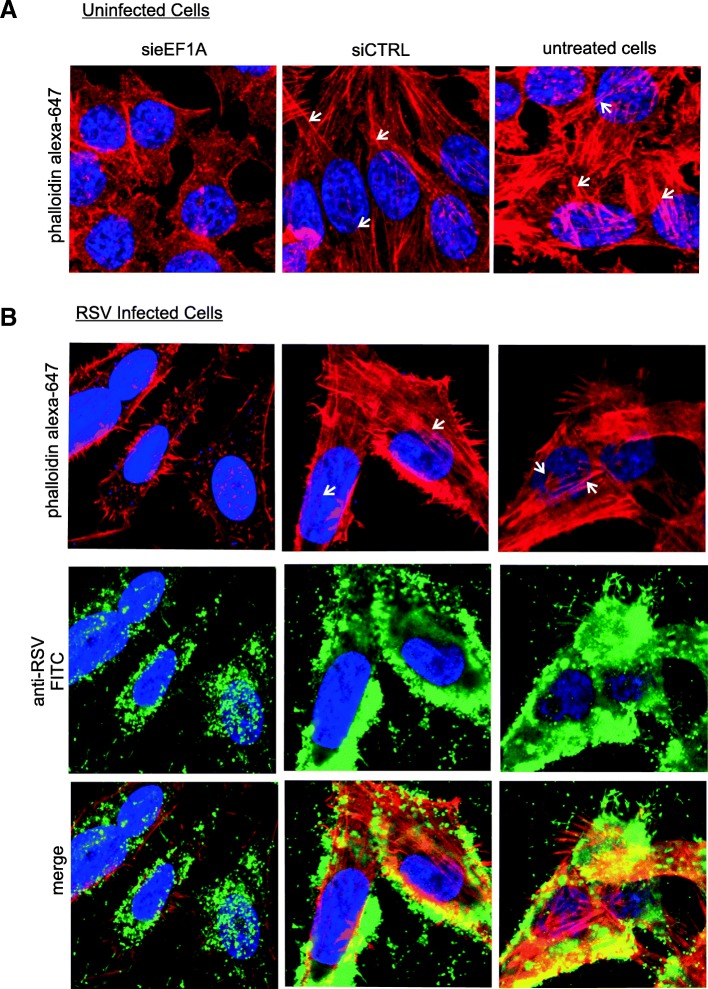
Downregulation of eEF1A levels by siRNA causes loss of actin stress fibers in uninfected and RSV infected HEp-2 cells. HEp-2 cells treated for 48 h with siRNA were (**a**) left uninfected or (**b**) infected with RSV. After 96 h p.t., the infected cells were fixed and stained with (**a** and **b**) Alexa Fluor 647 phalloidin and (**b**, second row) an anti-RSV polyclonal antibody, and counterstained with an appropriate secondary antibody conjugated to FITC. (**b**, third row) A merged image showing actin and RSV staining. Cells were imaged by confocal microscopy and a maximum intensity projection is shown. Examples of stress fibers in uninfected (**a**) and infected cells (**b**) are indicated by arrows

We previously demonstrated that eEF1A knock-down reduces RSV replication and subsequent shed virus [8]. Considering the role of actin in RSV trafficking and assembly [20] and the association between actin and eEF1A [21], here we examined if reduced infectious RSV release associated with knockdown of eEF1A is associated with actin structure changes. We performed experiments similar to those described previously [8], utilising transfection of siRNA to knock-down eEF1A (sieEF1A) followed by RSV infection. Firstly, eEF1A knock-down efficiency by sieEF1A in HEp-2 cells was confirmed at 24, 48 and 96 h p.t. compared with non-transfected and control siRNA (siCRTL) transfected cells (see Additional File 1A). Western blot results demonstrated that eEF1A expression was reduced at 24 h p.t., reached maximum reduction at 48 h p.t. (> 80% knock-down) and remained reduced at 96 h p.t.. Replicate transfected cells were fixed and stained to detect the distribution of F-actin at 48 h p.t. when the eEF1A knockdown was apparent. Knock-down of eEF1A caused a reduction in actin stress fiber formation and increased diffuse staining in the cytoplasm of uninfected cells (Fig. 1a, left panel). This is evident in comparison to cells transfected with a siRNA control or non-transfected, in which the long actin stress fibers spanning the cell are evident (Fig. 1a, middle and right panels, see arrows for examples). These results support an important role for eEF1A in actin stress fiber formation in HEp-2 cells.

We have previously demonstrated that knock down of eEF1A reduces infectious RSV release due to a reduction in genome replication [8]. Here we repeated those experiments and stained fixed cells to visualise actin in order to confirm that actin stress fiber levels are also reduced by eEF1A knock down in RSV-infected HEp-2 cells (Fig. 1b, left panels) compared to cells also infected with RSV but transfected with a siRNA control (siCTRL) or not transfected (Fig. 1b, middle and right panels). For example, actin stress fibers, such as those spanning the stained nuclei, are evident in control cells but not in sieEF1A-treated cells (Fig. 1b). An anti-RSV antibody stain showed that eEF1A knock-down reduced accumulation of viral proteins throughout the cell (Fig. 1b, third row) and formation of virus induced filopodia. Western blot confirmed eEF1A knock-down in replicate cells for these experiments (see Additional file 1B). This suggests that in addition to reduced RSV genome synthesis, reduced levels of eEF1A expression does effect actin stress fiber formation in RSV infected cells, which may also play a role in the reduction of infectious virus release.

### Did B treatment of HEp-2 cells reduces cellular stress fibers and reduces RSV egress

**Fig. 2:**
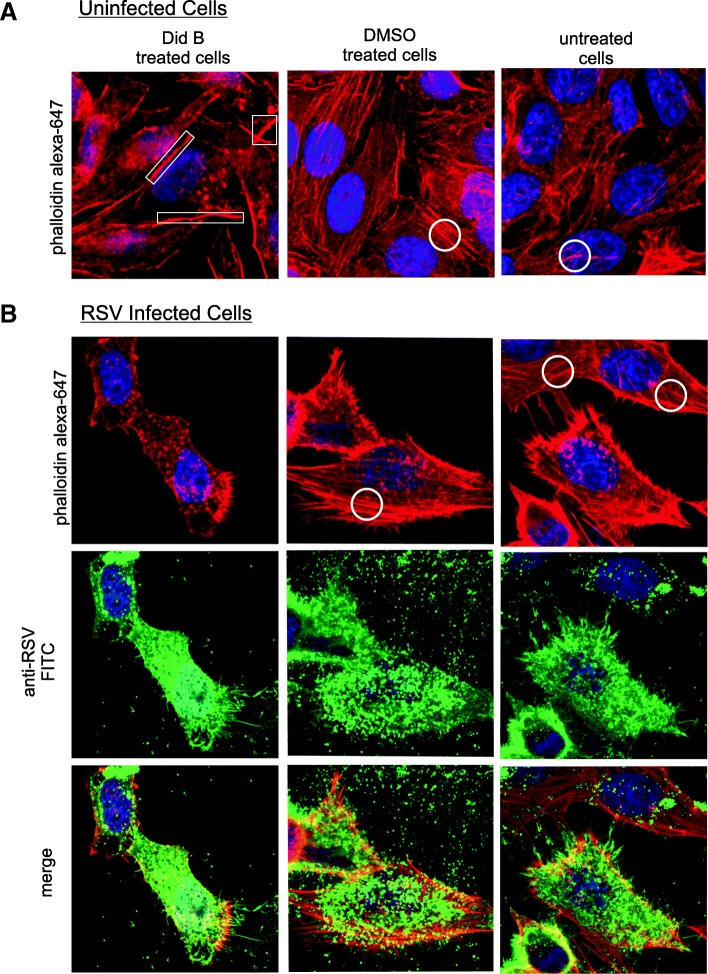
Did B treatment downregulates actin stress fiber formation in HEp-2 cells. HEp-2 cells were treated with 2.5 nM Did B, DMSO or left untreated. **a** Uninfected and (**b**) RSV infected were stained 48 h later to show (**a** and **b**) F-actin using Alexa Fluor 647 phalloidin or (**b**) RSV protein using a anti-RSV antibody. The stained cells were visualised by confocal microscopy and a maximum intensity projection is shown. **a** In Did B treated cells, examples of F-actin accumulation at the cell cortex are boxed. **a** and **b** Actin stress fibers, as exampled by circles, in control HEp-2 cells that are sharply downregulated in Did B treated cells. **b**, (bottom row) The merged images of Alexa Fluor 647 phalloidin and FITC staining are shown

**Fig. 3:**
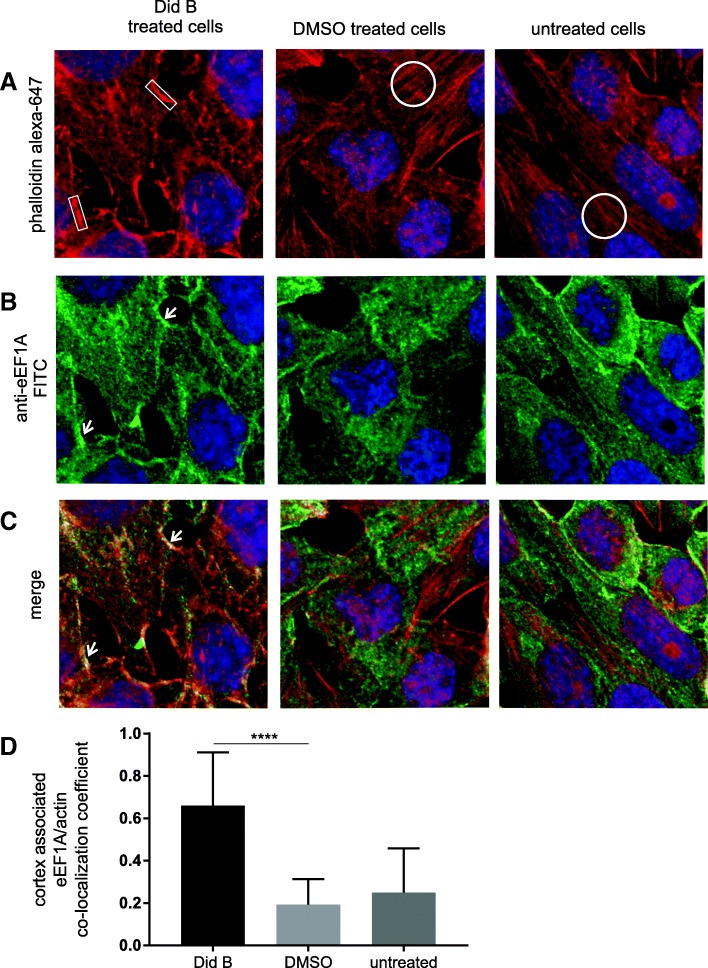
The effect of Did B treatment on F-actin and eEF1A distribution in HEp-2 cells. **a**-**c** HEp-2 cells were treated with Did B at 2.5 nM, DMSO or untreated as indicated. Fixed cells stained with Alexa Fluor 647 phalloidin or an anti-eEF1A antibody that was counter stained with a FITC conjugated secondary antibody. All images were acquired using identical settings and show a maximum intensity projection. Boxes highlight accumulation of F-actin at the cortex. Circles highlight examples of stress fibers. The arrows highlight regions of the plasma membrane with increase co-localization of eEF1A and F-actin, which is less evident in the control samples. Representative maximum intensity projection images are shown from an experiment performed three times with similar results. **d** The colocalization coefficient of eEF1A and actin at the cell cortex for untreated cells or those treated with DMSO or 2.5 mM Did B is shown. The cortex regions of at least 12 cells were measured. ZEN Blue Black software was used to analyze FITC (eEF1A) and phalloidin (actin) pixels using identical parameters. Colocalization analysis was performed on a pixel by pixel basis of z-stacks of images [19]. The correlation coefficient mean value and SD is shown. The **** symbols indicates a *P* values < 0.0001

To further examine the roles of eEF1A in actin structure and RSV production, we treated cells with Did B. Did B is a cyclic depsipeptide that can bind specifically to eEF1A and if present at sufficient levels can inhibit eEF1A’s function in protein translation [22]. A low level of Did B that does not inhibit translation is reported to inhibit HIV-1 replication [9]. First we checked if treatment with Did B affected cell viability and translation using serial dilutions of Did B and an established assay [9]. In HEp-2 cells, the results showed that concentrations of 4 nM Did B or less had no significant effect on luciferase levels after 48 h (see Additional file 2A). A MTS-based assay also showed that Did B had no significant adverse effect on cell viability at 4 nM or less after 48 h treatment (see Additional file 2B). To identify if Did B treatment had a similar effect on actin stress fiber formation as eEF1A knock-down, uninfected (Figs. 2a and 3) and RSV infected (Fig. 2b) HEp-2 cells were treated with 2.5 nM of Did B for 48 h. Actin fibers were detected via phalloidin staining (Figs. 2 and 3) and cells were additionally probed with an anti-eEF1A antibody (Fig. 3). A loss of actin stress fiber structures was observed in all cells treated with Did B (Figs. 2 and 3a, left panel) compared to a dimethyl sulfoxide (DMSO) carrier treated or untreated cells (where in Figs. 2a and 3 examples of actin bundles are circled). Did B treatment increased localisation of F-actin to the cortex (Fig. 2a and 3a, left panel, see boxes) and co-localization with eEF1A (Fig. 3) at the cortex as evidenced by the increase in yellow visible in the merged view (Fig. 3c, left panel, see arrows) compared to controls. Image analysis on a pixel by pixel basis of the cortex region of Did B treated cells compared to control cells showed an ~ 3 fold increased in colocalization of eEF1A with actin (Fig. 3d). The experiments show that 2.5 nM Did B avoided toxicity effects, downregulated stress fiber levels and increased accumulation and co-localization of F-actin and eEF1A at the cortex.

As a 2.5 nM Did B treatment could disrupt actin stress fiber formation, similar to the effect of eEF1A knockdown in HEp-2 cells (Fig. 1a) without reducing the overall level of eEF1A protein expression (Fig. 3b) (also see Additional file 2C and D), the effect of Did B treatment on RSV replication and release were examined. HEp-2 cells were treated with 2.5 nM of Did B at 4 h post-infection (p.i.) with RSV to investigate the effect on RSV replication and egress from cells at 48 h p.i.. F-actin staining confirmed that actin stress fibers were reduced in Did B treated cells (Fig. 2b, left panels), compared to DMSO-treated and untreated control cells (Fig. 2b, middle and right panels). RSV release from Did B treated cells was reduced, as demonstrated by an observable reduction in RSV within the extracellular spaces (Fig. 2b, middle row, left vs. center and right panels), an outcome consistently observed in samples. The combined data with eEF1A knock-down support a role for eEF1A in actin stress fiber formation and viral egress.

### Did B treatment slowed RSV-induced cell death and reduced RSV release

**Fig. 4:**
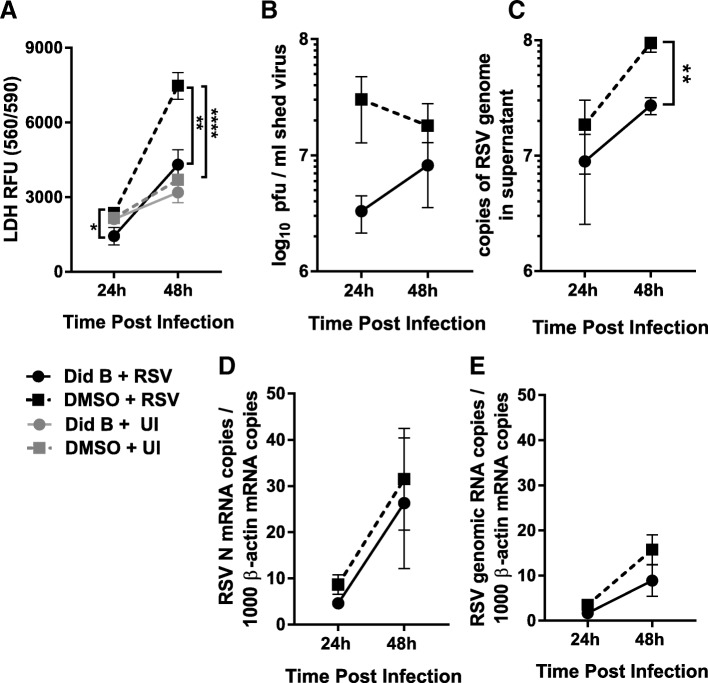
Did B treatment reduced RSV cytotoxic effects and virus release without affecting RNA genome replication or transcription. HEp-2 cells were infected with RSV at a MOI 1 and were treated 2 h later with 2.5 nM Did B, DMSO as a vehicle control or untreated. Cell culture supernatants were collected at 24 and 48 h p.i. **a** Cell death was quantified by lacto dehydrogenase (LDH) release. **b** The release of infectious virus was quantified using an immuno-plaque assay. **c** Viral genomic RNA released into the supernatant was quantified by RT-qPCR using primers to the RSV N gene quantified against a known copy number standard. **d** Total RNA was extracted from the same cells and RT-qPCR performed to quantify RSV N mRNA and (**e**) RSV genomic RNA. Copy number was calculated against a known copy number standard for both RSV and β-actin and expressed as a ratio of RSV/β-actin. Data are presented as the mean values of the experiments. The ** and **** symbols indicate *P* values of < 0.01 and < 0.0001 respectively

We further investigated the effect of Did B treatment on RSV replication and release, and also cell death p.i., by measuring shed virus, mRNA transcription and genome replication, and lactose dehydrogenase (LDH) release in cells treated with Did B compared to DMSO-treated cells at both 24 h and 48 h of p.i. Cells were infected with RSV at a multiplicity of infection (MOI) of 1 and then treated with Did B as previously detailed. Did B treatment protected cells from RSV-induced cell death, as there was significantly more LDH release from RSV-infected cells treated with DMSO than treated with Did B at both 24 and 48 h p.i. (Fig. 4a). The 2.5 nM Did B treatment did not induce cell death as there was no significant difference between LDH release from Did B treated and DMSO treated cells without RSV infection. This protection from RSV-induced cell death provided by Did B treatment was associated with reduced release of infectious RSV at both 24 and 48 h p.i. (Fig. 4b), as was also observed by anti-RSV staining of Did B-treated, RSV infected cells (Fig. 2b). The number of RSV genome copies detected in the cell culture supernatant was also reduced in Did B treated cells compared to DMSO treated cells, although this was only statistically significant at 48 h p.i. (Fig. 4c). Reduced cell death and reduced RSV production in cells treated with Did B supports the observation that Did B dysregulates cellular actin stress fibers in RSV-infected cells such that RSV egress is reduced (Fig. 2b). We were interested in whether this was a direct effect on release, or if Did B treatment affected RSV genome replication as we have observed previously in eEF1A knock-down experiments [8]. RSV transcription and replication were quantified by RT-qPCR of viral N mRNA (Fig. 4d) or genomic RNA in infected cells (Fig. 4e), as previously described [8]. Did B treatment did not significantly alter either RSV transcription or genome replication at either 24 h or 48 h p.i., however there was a trend towards lower genomic RNA at 48 h p.i. in Did B treated cells. The results suggest that at the concentration of 2.5 nM, Did B did not significantly affect RSV transcription and replication, especially at 24 h p.i., but significantly reduced infectious virus production and release, possibly a consequence of changes in actin stress fiber formation.

In summary, we provide evidence here that Did B, an eEF1A binding compound, significantly reduced eEF1A-induced actin stress fiber formation in HEp-2 cells. This suggests that Did B binding to eEF1A inhibits its actin bundling activity, which has not been previously reported. As actin and actin filaments play a key role in the formation and egress of RSV in infected cells, our data show that either decreasing cellular concentrations of eEF1A or targeting eEF1A with a small compound significantly affects RSV assembly and egress as well as reducing RSV-induced cytopathic effects.

## Discussion

Subversion of various host cell apparatus to promote viral replication and spread is a strategy used by all viruses. As the most abundant cellular protein, actin is a key player in many physiological processes [5]. Its arrangement within the cytoskeleton is vital to a plethora of dynamic cellular and structural functions including shape, cytoplasmic transportation, stabilization of organelles, cell movement and intracellular interactions. Consequently, viruses have evolved strategies to undermine the host cell’s actin-regulated functions to enable virus trafficking and egress [23]. Viruses in the family *Pneumoviridae*, such as RSV, require assembly within the cytoplasm, and dispersal of RSV virions via budding of the plasma. These activities are achieved through actin dynamics [11].

eEF1A is a major F-actin binding protein that has the ability to link F-actin into bundles forming structures such as stress fibers [5]. Given its abundance in the cell and its function in stabilising F-actin, binding to and facilitating F-actin bundling into stress fibers, it is not surprising that many viruses have evolved mechanisms to redirect cellular eEF1A as cofactors in viral transcription, translation and assembly [4, 7]. Previous studies have found eEF1A in highly purified fractions of a number of viruses, indicating that eEF1A may in fact be a critical component in the viral replication complex [4, 24]. Former research into the RNA virus, tomato bushy stunt virus (TBSV), demonstrated that a chemical inhibitor of eEF1A, Did B, effectively inhibited replication of TBSV in vitro [25].

Having previously demonstrated that RSV viral replication requires eEF1A dependant N-protein binding, we implicated eEF1A as a potential antiviral target for RSV [8]. Our aim in this current study was to investigate whether Did B could also mitigate RSV replication in vitro. By binding eEF1A, Did B blocks eEF1A’s role in translation reportedly by inducing a conformational change that blocks its interaction with eEF2 [17]. Our analysis has shown that treatment of HEp-2 cells with Did B at or below 4 nM does not significantly reduce the cell’s translation ability or viability. This is not entirely surprising as the estimated cellular concentrations of total eEF1A is high; approximately 40–60 μM, and ~ 80% of cellular eEF1A is bound to F-actin [26], which could mitigate an effect of a 2.5 nM concentration of Did B on translation but still affect its ability to stabilise F-actin stress fibers. When we treated these HEp-2 cells with either sieEF1A or 2.5 nM of Did B, a distinct loss of structure and organisation of actin stress fibers is evident within the cytoplasm. eEF1A knockdown and Did B affected F-actin and eEF1A distribution where increased accumulation near the plasma membrane was observed. This localization suggested a redistribution of F-actin to the cortex in cells that were treated with sieEF1A or 2.5 nM Did B.

Actin is known to play a significant role in viral entry for many viruses, particularly those targeting epithelial cells such as RSV [14, 27]. Actin is similarly essential for RSV egress, conventionally known to induce cell surface membrane budding in order to proliferate, which requires changes to the cytoskeleton [28, 29]. We have shown that knock-down of eEF1A disrupts cellular actin function in HEp-2 cells, as did Did B. Confocal images of HEp-2 cells treated with a concentration of 2.5 nM Did B and inoculated with RSV-A2 at an MOI of 1, present accumulated peripheral RSV particles and far less extracellular virions compared to control treated, RSV inoculated cells (Fig. 2). This suggests that the ability of RSV to modify the host cytoskeleton for release of newly replicated progeny is disrupted when eEF1A function in stress fiber formation is inhibited.

Our images of control treated HEp-2 cells display long, actin extensions in RSV infected cells and many filaments stained with an anti-RSV antibody; whereas cells treated with Did B appeared to have fewer extensions. This is in accord with filament formation observed in RSV induced A549 cells, as well as HMPV induced human bronchial epithelial cells (BEAS-2B) [14, 30]. These corresponding results indicate that pneumoviruses are capable of inducing actin-dependent cellular extensions in vitro in a range of cell lines.

We verified that cellular stress fibers are critical for RSV egress through a quantifiable reduction of extracellular RSV in HEp-2 cells treated with Did B. The number of infectious RSV particles released by Did B treated cells (shed virus) is considerably reduced at 24 h p.i., while the level of RSV genomic RNA in infected cells is similar to the cells with no treatment or DSMO treatment, indicating that the release of shed virus was affected in cells treated with Did B. The amount of cell death induced by RSV infection, quantified by LDH release, was significantly reduced in Did B treated cells at both 24 and 48 h p.i., compared to untreated cells. Taken together, these data suggests that by 48 h after inoculation with RSV, the quantity of viral particles released into the supernatant from Did B treated HEp-2 cells is reduced (Fig. 4), most likely as a consequence of the host cell’s inability to form actin structures formed through crosslinking of F-actin. The significantly elevated level of non-infectious RSV genomic RNA released from control cells (RSV infected, but not treated with Did B) at 48 h p.i., is likely due to more rapid cell death and the resultant release of a greater amount of RSV genomic RNA into the supernatant from necrotic cells. We propose that treatment with 2.5 nM of Did B, not only mitigates the release of RSV through budding or extracellular extension via disruption of actin bundling, but also delays cell death, in turn delaying the release of infectious RSV and reducing spread of progeny. Treatment with Did B at 2.5 nM did not affect transcription of RSV nucleocapsid mRNA or genomic RNA replication significantly compared to the control treatment up to 48 h p.i..

In light of our results, selective inhibitor of actin crosslinking by eEF1A could be a part of an antiviral strategy for pneumoviruses. Although complete inhibition of RSV was not observed in this study, reduced RSV-induced cell death was a noted consequence of actin stress fiber dysregulation in RSV infected cells. This Did B effect would be worth testing with other viral species.

## Additional files

Additional file 1: Western blot analysis of cell lysates prepared from cells treated as indicated. The relative level of eEF1A was normalised to β-tubulin in the same sample by analysis of digital images using ImageJ software. The experiments were repeated three times with similar results and representative results are shown. (PDF 444 kb)

Additional file 2: The effect of various Did B concentrations on HEp-2a translation, cell viability and levels of eEF1A. (A) HEp-2a cells were transfected with a Gaussia luciferase reporter plasmid and then treated with serial dilutions of Did B using 1 nM to 16 nM concentrations as indicated for 48 h. The level of Gaussia luciferase made by the transfected cells was measured. (B) An MTS cell viability assay was performed on HEp-2a cells treated with the same concentrations of Did B. Both experiments were performed in triplicate and repeated twice. (C) Western blot of cell lysates from untreated, DMSO or Did B treated HEp-2a cells that were incubated for 24 or 48 h as indicated. The blots were stained with either anti-eEF1A or anti-β-tubulin and a secondary antibody conjugated to HRP. (D) The digital images from three independent experiments were analyzed using ImageJ software. The relative eEF1A signal was normalised to the level of β-tubulin detected on the same blot for each sample. The graph shows mean value and standard deviation for the experiments. Where indicated, n.s. indicates that a Student’s T-test determined that the mean values were not significantly different. The mean values and standard deviation of the results are shown. The n.s. indicates that a Student’s T-test determined that the difference between the samples compared was not significant. (PDF 461 kb)

## Abbreviations

ARP: Actin-related protein
Did B: Didemnin B
DMSO: Dimethyl sulfoxide
eEF1A: Eukaryotic translation elongation factor 1A
F-actin: Filamentous actin
MOI: Multiplicity of infection
p.i.: Post-infection
p.t.: Post transfection
RSV: Respiratory syncytial virus
