# Supplementary material for: The eukaryotic translation elongation factor 1A regulation of actin stress fibers is important for infectious RSV production
Source: Virol J. 2018 Nov 26;15:182. doi: 10.1186/s12985-018-1091-7 (PMC6260765; doi:10.1186/s12985-018-1091-7)
Supplement: Supplementary file 1 — Western blot analysis of cell lysates prepared from cells treated as indicated. The relative level of eEF1A was normalised to β-tubulin in the same sample by analysis of digital images using ImageJ software. The experiments were repeated three times with similar results and representative results are shown. (PDF 444 kb) [file 12985_2018_1091_MOESM1_ESM.pdf]

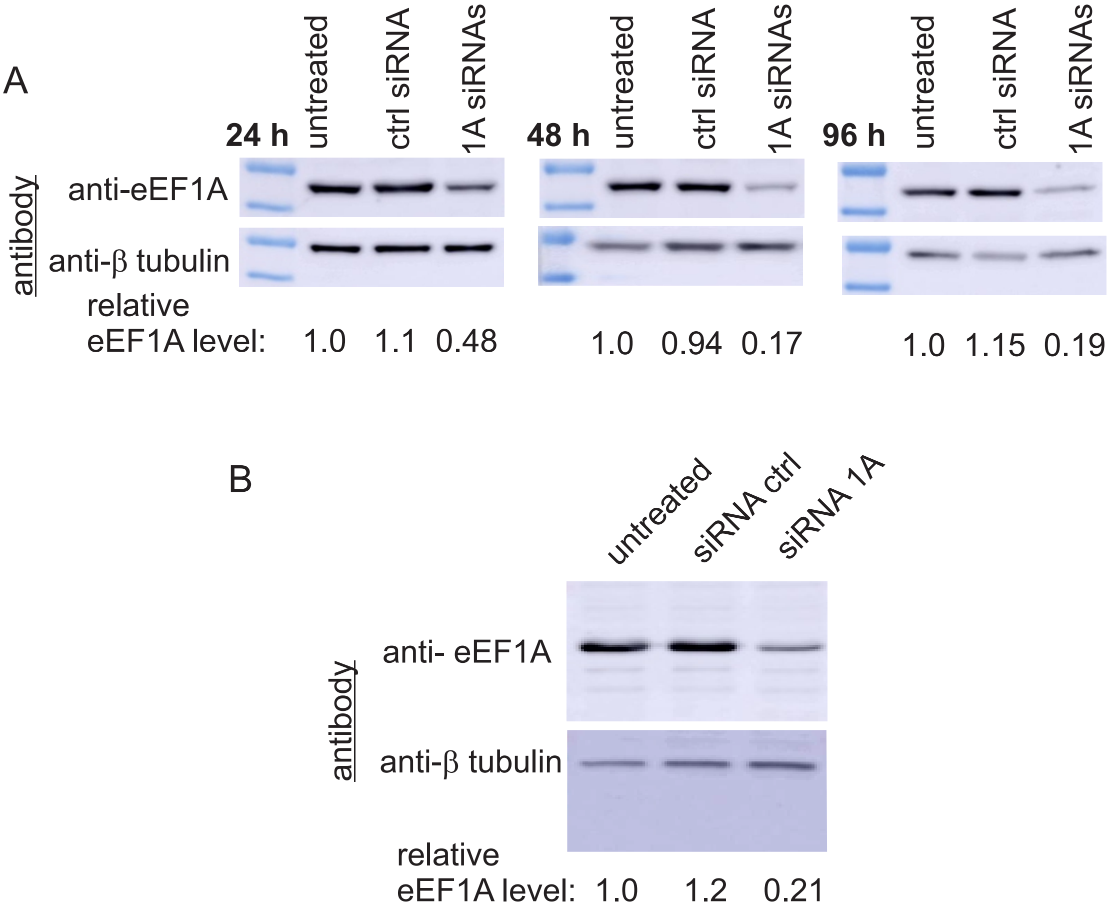

Additional file 1. Western blot analysis of cell lysates prepared from cells treated as indicated. The relative level of eEF1A was normalised to β-tubulin in the same sample by analysis of digital images using ImageJ software. The experiments were repeated three times with similar results and representative results are shown.
