# Supplementary material for: The eukaryotic translation elongation factor 1A regulation of actin stress fibers is important for infectious RSV production
Source: Virol J. 2018 Nov 26;15:182. doi: 10.1186/s12985-018-1091-7 (PMC6260765; doi:10.1186/s12985-018-1091-7)
Supplement: Supplementary file 2 — The effect of various Did B concentrations on HEp-2a translation, cell viability and levels of eEF1A. (A) HEp-2a cells were transfected with a Gaussia luciferase reporter plasmid and then treated with serial dilutions of Did B using 1 nM to 16 nM concentrations as indicated for 48 h. The level of Gaussia luciferase made by the transfected cells was measured. (B) An MTS cell viability assay was performed on HEp-2a cells treated with the same concentrations of Did B. Both experiments were performed in triplicate and repeated twice. (C) Western blot of cell lysates from untreated, DMSO or Did B treated HEp-2a cells that were incubated for 24 or 48 h as indicated. The blots were stained with either anti-eEF1A or anti-β-tubulin and a secondary antibody conjugated to HRP. (D) The digital images from three independent experiments were analyzed using ImageJ software. The relative eEF1A signal was normalised to the level of β-tubulin detected on the same blot for each sample. The graph shows mean value and standard deviation for the experiments. Where indicated, n.s. indicates that a Student’s T-test determined that the mean values were not significantly different. The mean values and standard deviation of the results are shown. The n.s. indicates that a Student’s T-test determined that the difference between the samples compared was not significant. (PDF 461 kb) [file 12985_2018_1091_MOESM2_ESM.pdf]

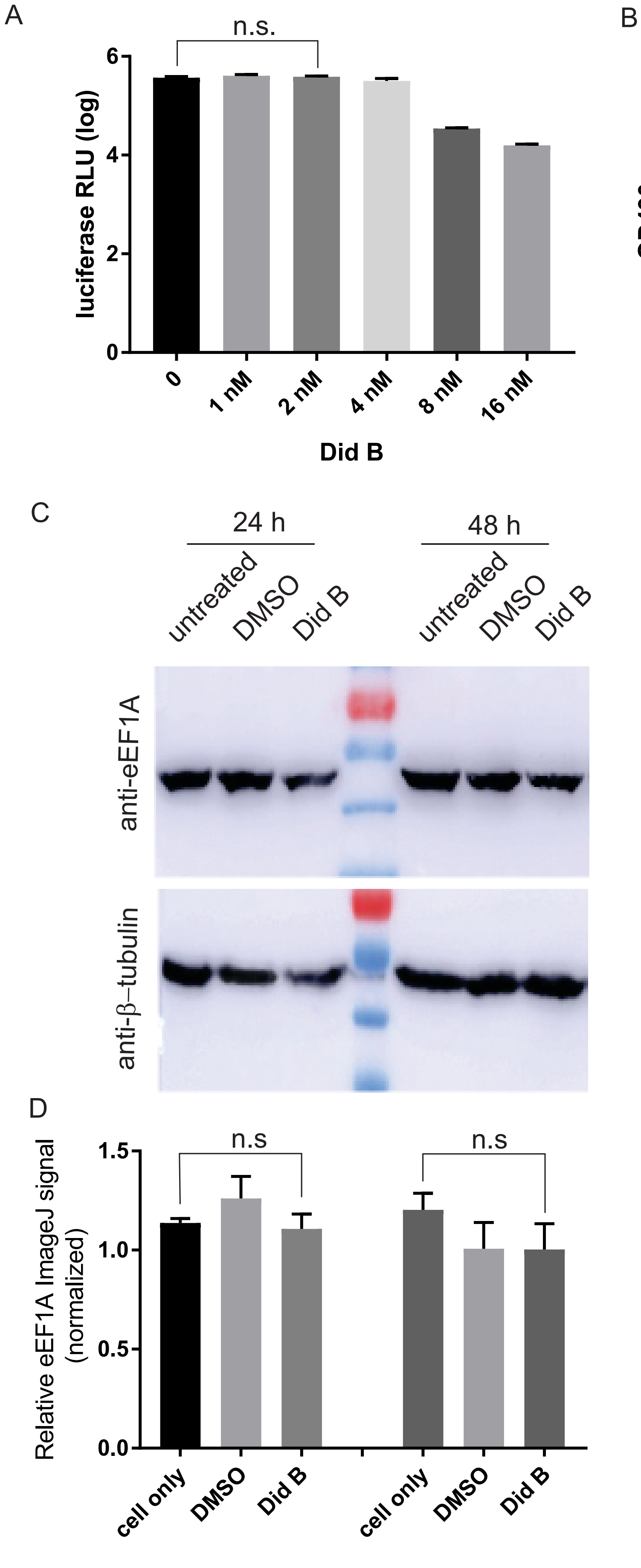

Additional file 2. The effect of various Did B concentrations on HEp-2a translation, cell viability and levels of eEF1A. (A) HEp-2a cells were transfected with a Gaussia luciferase reporter plasmid and then treated with serial dilutions of Did B using 1 nM to 16 nM concentrations as indicated for 48 h. The level of Gaussia luciferase made by the transfected cells was measured. (B) An MTS cell viability assay was performed on HEp-2a cells treated with the same concentrations of Did B. Both experiments were performed in triplicate and repeated twice. (C) Western blot of cell lysates from untreated, DMSO or Did B treated HEp-2a cells that were incubated for 24 or 48 h as indicated. The blots were stained with either anti-eEF1A or anti-β-tubulin and a secondary antibody conjugated to HRP. (D) The digital images from three independent experiments were analyzed using ImageJ software. The relative eEF1A signal was normalised to the level of β-tubulin detected on the same blot for each sample. The graph shows mean value and standard deviation for the experiments. Where indicated, n.s. indicates that a Student's T-test determined that the mean values were not significantly different. The mean values and standard deviation of the results are shown. The n.s. indicates that a Student's T-test determined that the difference between the samples compared was not significant.
